# Aneurysm

**Published:** 1908-05-02

**Authors:** 


### Points in Surgery.

### ANEURYSM.

### TREATMENT BY LIGATURE.

In the last article of this series the constitutional treatment of aneurysm was discussed, a term which includes all the general measures which may be adopted in the hope of a spontaneous cure following, or may be regarded as accessory to the interference of the surgeon, or lastly may be relied on alone when from the nature of the case operative measures are either inadvisable or not practicable.

### Extirpation of the Sac.

For there is no question that the proper treatment, whenever it is possible, is operation, and that of all measures hitherto devised some form of ligature is both the simplest and the most efficient. Some aneurysms may be completely extirpated after ligature of the trunk from which they spring above and below the tumour. This is, of course, the ideal treatment, but unfortunately it is but rarely possible. The manœuvre is most readily performed in traumatic aneurysms, since in these the vessel is generally a superficial one—*e.g.* the radial—and such an aneurysm rarely attains to large dimensions. Several cases, however, have been reported from America in which popliteal aneurysms have been extirpated and the divided ends of the artery have been united by a method analogous to end-to-end anastomosis of the intestine. The name of arteriorrhaphy has been applied to this manœuvre. Even apart from the difficulty of getting the cut ends into apposition (which must be very great if the aneurysm is a large one), and the risk of reactionary hæmorrhage owing to the line of suture giving way, disadvantages of this operation are readily imagined. For one has an artery with a line of sutures which must be a weak spot, even if the immediate result of the anastomosis is successful, and at the same time the presence of all the conditions which produced the original aneurysm—*e.g.* let us say, increased blood pressure. The operation does nothing towards getting rid of this last factor, and it seems only reasonable to infer that the probability is that a second aneurysm will occur *in situ*. And again, it seems an unnecessary step to unite artificially the cut ends of the vessel when nature is ready to produce an excellent collateral circulation if these are merely ligatured. Much as one may admire the ingenuity of such surgical methods, one cannot honestly regard them as sound.

### The Various Forms of Ligature.

Short of extirpation of the sac, some form of ligature is the next step to be considered; and the various forms of ligature are associated with historical names, since, as has already been said, aneurysm was one of the commonest of surgical maladies in times gone by, and many famous surgeons in succeeding generations turned their attention to the treatment of the condition.

Thus ligature of the vessel above and below, leaving the sac *in situ*, is attributed to Antyllus; whereas ligature of the proximal part of the vessel only is called after Anel, who first practised the method in a case of aneurysm of the brachial artery in 1710.

John Hunter suggested in 1785 that the best place to ligature the vessel was not immediately above the swelling, but above the first collateral branch. He claimed that his operation had several advantages over Anel's, namely (1) that it was easier to tie the vessel at some distance; (2) that the vessel-wall was less likely to be unhealthy in this situation; and (3) that there was less chance of injuring the sac-wall during the necessary manipulations. His first argument still holds good, and so partly does his third. But it must be remembered that the surgeons of those days were much more terrified of hæmorrhage than we are, for the simple, ingenious, and efficient hæmostatic pressure forceps invented by Sir Spencer Wells, which are now an indispensable adjunct to almost every surgical operation, was unknown to them.

The first argument that it is easier to tie the vessels at some distance from the aneurysm remains as true to-day as it ever was. For Hunter's original operation was ligature of the superficial femoral artery for a popliteal aneurysm; and in this operation it is more true than in any other, since a popliteal aneurysm enlarges upwards until its upper pole rests against the adductor magnus muscle, so that it is often impossible to apply a ligature between the muscle and the aneurysm.

The object of proximal ligature is in all cases the same, whether Anel's method or Hunter's be employed, and that is to remove the force of the circulation and so allow the blood contained in the sac to coagulate. An aneurysm can only be regarded as cured when the main trunk from which it springs has been occluded and the blood in the sac has subsequently clotted and this clot has spread up the parent stem as far as the ligature.

If proximal ligature is impossible, the vessel may be ligatured on its distal aspect. Two operations with this object are described: (1) Brasdor's, in which the trunk of the vessel, and (2) Wardrop's, in which only the terminal branches are tied. Neither of these is, as might be expected, so successful as proximal ligature.

Several risks attend the operation, though not so many as did in the old days when surgical interference was always attended by sepsis, so that secondary hæmorrhage is, or should be, rare; but ligature of the main vessel of a limb may, and sometimes does, lead to gangrene. Therefore, after the operation, everything must be done to help the development of the collateral circulation. The limb must be wrapped in wool and elevated; and the extremities must be watched for any change in colour or in temperature, and gentle friction should be applied to them if this makes its appearance.

